# Recombinant Cytokines from Plants

**DOI:** 10.3390/ijms12063536

**Published:** 2011-06-03

**Authors:** Agnieszka Sirko, Tomas Vaněk, Anna Góra-Sochacka, Patrycja Redkiewicz

**Affiliations:** 1 Institute of Biochemistry and Biophysics Polish Academy of Sciences, ul. Pawinskiego 5a, 02-106 Warsaw, Poland; E-Mails: annag@ibb.waw.pl (A.G.-S.); PatiRed@ibb.waw.pl (P.R.); 2 Laboratory of Plant Biotechnologies, Joint Laboratory of Institute of Experimental Botany and Institute of Crop Research, Prague, Czech Republic; E-Mail: vanek@ueb.cas.cz

**Keywords:** cytokines, pharmaceutical proteins, plant-based production systems, molecular farming, interleukins, transgenic plants

## Abstract

Plant-based platforms have been successfully applied for the last two decades for the efficient production of pharmaceutical proteins. The number of commercialized products biomanufactured in plants is, however, rather discouraging. Cytokines are small glycosylated polypeptides used in the treatment of cancer, immune disorders and various other related diseases. Because the clinical use of cytokines is limited by high production costs they are good candidates for plant-made pharmaceuticals. Several research groups explored the possibilities of cost-effective production of animal cytokines in plant systems. This review summarizes recent advances in this field.

## 1. Introduction

Development of DNA recombination and plant transformation techniques resulted in creating the novel protein production platforms based on either whole plants or plant cells. The results of the first experiments describing the plant-based production of pharmaceutical proteins were published about 25 years ago. The successful production of the human growth hormone in tobacco and sunflower [1] and of albumin in tobacco and potato [2] indicated that plant-based production systems might be used for the production of mammalian proteins. The process of using plant-based systems as highly effective production platforms for the molecules with biotechnological (industrial or pharmaceutical) significance is named molecular farming, while the pharmaceutical products obtained in the plant-based systems are often called plant-made pharmaceuticals (PMPs). At present, a list of PMPs in various stages of development or potential commercialization is quite extensive and includes various antibodies and their fragments, vaccine antigens, blood substitutes, enzymes, cytokines and other important and valuable proteins. Multiple examples of such proteins and the extensive evaluation of the advantages and disadvantages of various plant-based platforms for the expression of the particular targets can be found in many recent reviews (for example, [3], see also below), therefore this work is limited only to a small group of PMPs, the cytokines.

## 2. Cytokines and Their Therapeutic Application

**Figure 1:**
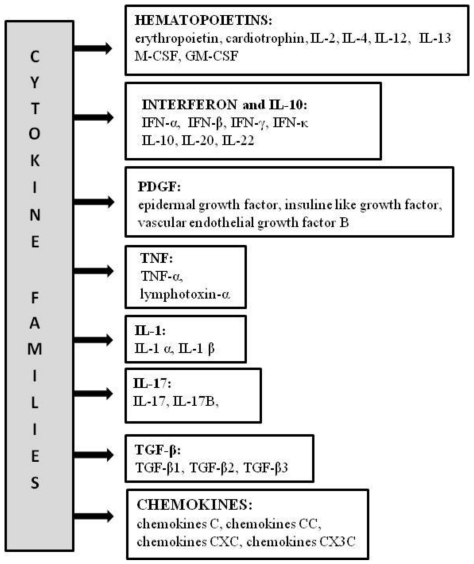
The families of cytokines (according to the Kyoto Encyclopedia of Genes and Genomes [4]).

Cytokines are small polypeptides, proteins or glycoproteins involved in the regulation of processes as diverse as proliferation, differentiation and mobility of cells. Cytokines are important components of the immune system; however they also participate in embryogenesis, affect the hematopoietic system and act on neuronal cells. The anatomic and structural distinctions between hormones and cytokines are unclear and cytokines are sometimes named the hormones of immune system. They can have an affect not only on cells in the close proximity but also those in distant organs. Cytokines are characterized by a considerable complexity of actions such as redundancy, pleiotropy, multifunctionality, synergistic or antagonistic effects and cascades of positive or negative feedbacks. At present, more than 100 different cytokines are known. Several methods of division of this large family into subgroups have been used. The most convenient seems to be functional categorization according to KEGG (Koto Encyclopedia of Genes and Genomes [4]). As shown in Figure 1, at least 8 families can be distinguished: Class I cytokines (hematopoietin family), Class II cytokines (interferons/IL-10 family), platelet-derived growth factors (PDGF), tumor necrosis factors (TNF family), IL-1 family, IL-17 family, tumor growth factor family (TGF-beta family) and chemokines. For more information about the cytokines the reader is referred to the Cytokines & Cells Online Pathfinder Encyclopedia (COPE) [5].

An imbalance in cytokine production or signaling contributes to various pathological immune and inflammatory disorders. In addition, plasma levels of various cytokines may give information on the presence, or even predictive value of inflammatory processes involved in autoimmune diseases such as rheumatoid arthritis. The biological role of cytokines and their dual (immunosuppressive as well as immunostimulatory) properties are a strong indication for multiple clinical applications. A number of recombinant cytokines have been approved for clinical use (Table 1) and multiple cytokine therapies are in clinical trials [6–8]. Not only the cytokines but also cytokine receptors, antagonists of the cytokine receptors and the relevant specific antibodies can be used in therapy [8]. Significant numbers of cytokines have been tested as potential adjuvants of immunological response, particularly during mucosal immunizations, such as oral, intranasal and intravaginal [2,9]. Granulocyte-macrophage colony stimulating factor (GM-CSF) seems to e one of the best candidates for PMP because it is relatively stable, consists of one kind of polypeptide, *i.e*., it is encoded by one gene, and, essentially, it is well tolerated by the patients. Numerous new implications of this cytokine have been proposed and tested in preclinical and clinical trials, including the combined application of GM-CSF and IL-2 as adjuvants in cutaneous melanoma along with autologous vaccine [10]. However, it is necessary to mention that the use of cytokines as adjuvants in vaccines is considered controversial because they can sometimes have serious side effects such as vascular leak syndrome [11]. An interesting idea is the production and application of “fusokines”, hybrid molecules generated after cloning and fusing two separate cytokine encoding cDNAs into a single open reading frame, however these experiments have been limited to the murine experimental models [12].

## 3. Recombinant Cytokines from Plant-Based Platforms

Plant-based production of recombinant cytokines is an emerging area and most previous research has concentrated on a few well-characterized cytokines. Some most interesting examples from the list shown in Table 2 are briefly discussed below.

### 3.1. Hematopoietin Family

Erythropoietin was one of the first cytokines produced in plant cells [13]. The cDNA encoding human erythropoietin was expressed from the 35S promoter of cauliflower mosaic virus (CaMV) in the tobacco BY2 cells. The signal peptide at the *N*-terminus was intended to enable the extracellular secretion of the recombinant protein. The secretion was observed in the protoplasts cultures, while in the cell cultures erythropoietin was translocated though the cellular membrane but it stayed bound by the cell wall and was not secreted into the medium. Most probably, the size of 30 kDa was too large for free migration through the cell wall. The protein is glycosylated in animal cells, so was examined in plant cells, however the pattern and length of the sugar chain were different. Unfortunately, only a low yield (0.0026% of total soluble protein [TSP]) was achieved. The protein was biologically active *in vitro* but not *in vivo*. The possible explanation for this might be the difference in glycosylation, which is known to affect the protein stability in blood.

Several independent groups focus on the production of human GM-CSF. Initially, it was produced in tobacco [15,16] and rice [17–20] cell suspension cultures. One of the most interesting approaches to improve the yield was the addition of either mineral salts or BSA to the growth medium, which stabilized the secreted cytokine [15]. A significant (up to 4-fold) increase of the yield was achieved due to the addition of 5% (w/v) gelatin to the medium [16]. However, in cultures older than 4 days, gelatin inhibited cell growth, presumably by activation of proteolytic enzymes. Much higher efficiency was achieved in cultures of rice cell suspension, particularly with the specific promoter of rice amylase, Ramy3D [17]. Using this system, a yield of 129 mg/L of culture was achieved in the absence of sugar (when this promoter is activated). The system was subsequently optimized in three strategies: (i) silencing of the gene encoding a-amylase, a dominant protein secreted by rice cells [18]; (ii) silencing of the gene encoding cysteine proteinase secreted by the rice cells to the medium [19]; and (iii) co-expression of cytokine with the protease inhibitor [20]. Each of these strategies increased the yield of recombinant GM-CSF at least two-fold.

Whole plant platforms were also used for GM-CSF production. The maximal yield in the leaves of transgenic sugarcane was about 0.02% [21] and in tobacco leaves about 0.22% of TSP [25]. In the seeds of transgenic rice recombinant GM-CSF accumulated up to 1.3% of TSP [23]. The highest expression (up to 2% TSP) was reported for the viral vector based on PVX (potato virus X) with modified coat protein [26]. In most cases the biological activity of recombinant GM-CSF was confirmed *in vitro*, while in at least in one case it was also verified *in vivo* in a mouse model [24].

The first report about the expression of recombinant IL-2 and IL-4 in tobacco cell suspension cultures was published in 1998 [27]. The authors assumed that the presence of intrinsic signal peptide would provide an efficient extracellular secretion of recombinant proteins. In fact, most of the cytokine produced was retained in the cells and only the secreted proteins had biological activity. Other strategies include targeting of the recombinant IL-4 proteins into endoplasmic reticulum, using the promoter specific to potato tubers or expression as a protein fusion with elastin, however the reported yield was never higher than 0.08% for IL-4 [30] or 115 U/g of potato tuber for IL-2 [28].

Other examples of cytokines from this group produced in plant-based platforms include cardiotrophin 1 [41], interleukin 12 [35–38], interleukin 13 [39] and interleukin 18 [40]. Interestingly, IL-12 was produced as a multimeric protein at levels exceeding 5% of TSP after coinfiltration of *Nicotiana benthamiana* leaves with two Agrobacterium strains individually encoding each subunit [55]. Cardiotrophin 1 was produced in transplastomic tobacco plants with an efficiency of about 5%. In addition, it appeared that its biological activity is inhibited by light, which most probably destroys the protein conformation [41].

### 3.2. Interferon and IL-10 Family

Interferons have been frequently selected as candidates for production in plant-based systems. The first reports about the plan-based production of human interferon date back to the beginning of the 90 s [56], however it was the usage of transplanstomic plants that allowed for the huge increase of yield, up to 20% of TSP [45]. It is worth mentioning that not only human interferons but also interferons from chicken [49] and salmon [50] were successfully produced in plant-based systems.

In plant-based systems, interleukin 10 was targeted either to chloroplasts or mitochondria, with better results for the former organelle [32]. In the case of this cytokine, the best yield of 0.27% TSP was achieved after expression of IL-10 in fusion with elastin [30].

### 3.3. Other Families

The single representatives of the other families of cytokine were produced in plant systems (Table 2).

Tumor necrosis factor (TNF-α), which is a member of the TNF family, has been produced in potatoes [51]. The authors used two types of expression cassettes, both containing 35S promoter of CaMV and the translation enhancer from TMV. One of the cassettes had additional sequences encoding the *N*-terminal signal peptide and the *C*-terminal SEQDEL peptide, responsible for ER targeting. However, this strategy did not result in any significant increase of cytokine accumulation. In both cases the yield was similar and reached about 15 μg of biologically active TNF-α per 1 g of plant tissue.

Interleukin 18 belongs to the IL-1 family. In this case, the enhanced 35S promoter and the translation enhancer from TMV were used; however the yield was moderate, up to 0.05% of TSP [40].

Bioactive human fibroblast growth factor 8b (FGF), a member of PDGF family, was produced in tobacco plants [52]. The cDNA coding hFGF8b was cloned under control of the double CaMV 35S promoter (CaMV35SS). In an Agrobacterium-mediated transient expression system after vacuum leaves infiltration the yield of the Ni-NTA affinity chromatography purified proteins c-myc-His tagged FGF8b and His-KDEL tagged FGF8b was 2.7% and 4.1% of TSP (90 and 150 μg/g FW), respectively.

Another member of the same PDGF family, Insulin-like Growth Factor 1 (IGF-1), was efficiently produced in transplastomic plants [53]. Cell proliferation assays in human HU-3 cells demonstrated the biological activity of this recombinant protein.

## 4. Prospects for Commercialization Plant-Produced Cytokines

The successful introduction of plant-produced recombinant cytokines into the market might encounter problems similar to those encountered in the commercialization of any other PMPs [57]. These problems can be divided into two main categories: (i) technological (such as low yield, poor performance of the recombinant product or problems with postharvest bioprocessing) making the platforms economically uncompetitive and (ii) legal (such as public objections and stringent regulatory requirements for open-field cultivation of transgenic plants, high regulatory-approval costs and long timelines), which result in a long timeframe-to-market and a lack of interest from the potential investors.

### 4.1. Strategies Used to Improve the Performance of Plant-Base Production Platforms

The researchers dealing with molecular farming issues have always been aware of the needs for technological improvement. Various factors limit the yield of PMPs and there are multiple possibilities to overcome the yield and economic constrains. These problems have been frequently considered in many excellent reviews [58–60]. The concerns generally expressed with regard to PMPs also pertain to the plant-produced cytokines. Therefore, we would like to mention briefly only a few strategies, which were successfully applied in the case of this type of therapeutics. First of all, some PMPs can be produced in edible crops to eliminate the need for purification of the desired products in the case of oral application of the recombinant proteins. In addition, several critical elements must be optimized to get a high yield of PMP, including the use of tissue specific promoter or optimization of the codons within the transgene to match the optimal codons of the host. For example, efficient accumulation Insulin-like Growth Factor I (IGF) was achieved in transgenic tobacco chloroplasts after codon optimization of the transgene [34,53]. The native IGF gene (IGF-n) with less than optimal for chloroplast AT content of 41% or synthetic (IGF-s) gene with optimized 60% AT were cloned into a vector containing the *psbA* promoter, 5′UTR (which enhances translation under illumination) and 3′UTR (which increases the stability of the transcript). The IGF-n transgenic plants had an expression level of 9.5% TSP. In IGF-s plants, expression level increased to 11.3% TSP, however the expression of IGF was increased up to 32% TSP under continuous illumination by the chloroplast light regulatory elements. The importance of protein stability is illustrated by other reported cases, where significant elevation of the yield was achieved by targeting the PMP into the storage organs such as seeds or grains [22–24,33]. For example, a strong endosperm-specific promoter (*Gt13a*) and rice-preferred codons optimization improved the transcription and translation of hGM-CSF, giving the yield of 14 μg/seed [24]. In this case also a signal peptide that targeted recombinant protein into the endomembrane system of rice endosperm such as the ER and protein bodies was used to protect against degradation by proteases. Additionally, it is worth mentioning that accumulation in seeds might eliminate the need for low temperature maintenance during transport and long-term storage of PMPs. There are also successful examples of fusion strategies being used to obtain the high yield of recombinant cytokines in plants, including IGF-1 produced in fusion with the *C*-terminus of a rice luminal binding protein [54]. The appropriately designed fusion, which does not interfere with the biological activity of the recombinant proteins, might be a safe and effective oral delivery system for cytokins in general.

Proteomic analysis of the effects of massive over-accumulation of GM-CSF in plants gave us some information about the fate of recombinant proteins in plant cells [61]. This work also indicated that the presence of recombinant protein might affect the total proteome of grain cells. For example, the major endogenous storage proteins, glutelin, globulin and prolamin, and a majority of carbohydrate-related proteins were down-regulated, while 26S proteasome-related proteins and molecular chaperons were up-regulated in transgenic rice endosperm. The author concluded that over expression of recombinant proteins induced unfolded protein response and that overall protein trafficking in rice endosperm was affected. This work shows that the influence of transgene expression on host metabolism is an important issue.

### 4.2. Other Problems to Consider

The initial pictures describing the feasibility of the production of edible plant-made pharmaceuticals or vaccines in carrots or bananas appeared somewhat naive. A direct consumption (oral application) of plant parts containing recombinant therapeutics might be controversial because of the need to meet the existing rigorous regulations concerning consistent composition of medicines for humans. Most legal and regulatory issues linked to the commercialization of PMPs were extensively discussed in a number of recent reviews [57,62,63] and they will not be repeated here. There are also other problems and questions regarding the use of genetically modified plants and their effects on the human health and the environment in general. These issues include some concerns regarding ethics, related to the risk of transferring transgene (especially antibiotic-resistance markers) from genetically modified plants to the environment, gut microflora and pathogenic microbes. As mentioned earlier, the risk of uncontrolled spreading of the transgene can be substantially reduced by the strategy of biocontainment or physical containment. In addition, using marker-free technology eliminates the risk of transfer of the marker genes [64,65]. There are also some religious issues related to the consumption of transgenic plants with animal genes introduced into them, especially, for some strict vegetarian people and some ethnical groups with certain food preferences and restrictions, but this concern is outside the scope of the present review.

It is necessary to mention that the avoidance of plant cultivation in open areas, applying various approaches of containment use and the usage of only isolated (purified) recombinant products lead, of course, to more expensive products and limit the direct use of GM plants as edible sources of recombinant therapeutics.

## 5. Conclusions

Recent development of knowledge about the immune system opens new perspectives for the application of cytokines and opens a potentially large market, however finding the niche for plant-produced recombinant cytokines in the pharmaceutical market might be tough. Many technical and regulatory challenges limit the availability of this sector for the prospective investors. All molecular farming projects, including those involved in the production of therapeutics for the treatment of immune system disorders, must consider the costs and feasibility of standardization, validation and licensing of the potential medical products. The biopharming industry is in a dynamic state and many companies involved in plant-based biomanufacturing of recombinant proteins cease to exist before the commercialization of any products [57]. Nevertheless, there are now about 20 PMPs, including at least one cytokine (interferon α) in development as potential products for the pharmaceutical market [66].

## Figures and Tables

**Table 1:** Examples of cytokines approved for use in humans. Modified from [7,8].

Cytokine	Disease or Indication	Drug Name (Company)
G-CSF	Neutropenia	Neupogen/Filgrastim (Hoffmann-La Roche)
GM-CSF	Leukemia Bone marrow Stem cell transplants	Leukine/Sargramostin (Bayer)
Interferon-α (INF-α)	Chronic hepatitis B Chronic hepatitis C Hairy cell leukemia Chronic myeloid leukemia Condyloma acuminate AIDS-related Kaposi’s sarcoma genital warts	Intron A (Schering Plough) Roferon A (Hoffman La Roche) Infergen (Three Rivers Pharmaceuticals) Alferon N (HEMISPHERx Biopharma) Pegasys (Genentech USA/Roche) Pegintron (Merck)
Interferon-β (INF-β)	Relapsing multiple sclerosis	Betaseron (Bayer) Avonex (Biogen Idec)
Interferon-γ (INF-γ)	Malignant osteopetrosis Chronic granulomatous disease	Actimmune (Intermune Pharma)
Erythropoietin-α (EPO-α)	Anemia due to chronic renal failure HIV infected patients Chemotherapy Primary bone marrow disorders	Eprex (Cilag Jansen) Epogen (Amgen) Procrit (Ortho Biotech)
IL-2	Metastatic renal cell cancer Metastatic melanoma	Aldesleukin (Novartis) Proleukin (Prometheus Laboratories)
IL-11	Thrombocytopenia	Oprelvekin/Neumega (Genetics Institute, Inc./Wyeth/Pfizer)

**Table 2:** Examples of cytokines produced in plant-based systems.

Cytokine *	Method/Plant Material	Selected Elements of the Expression Cassette	Expression Level/Yield	Reference
erythropoietin	T/tobacco (BY2) cells suspension	35S promoter and terminator	0.0026% TSP; 25 pg/L	[13]
G-CSF	T/tobacco cells suspension	35S promoter with the double enhancer, Ω-translation enhancer; *nos* terminator	105 μg/L	[14]
GM-CSF	T/tobacco cells suspension	35S promoter, translation enhancer from TEV, His tag, T7 terminator	150 μg/L (intercellular); 250 μg/L (secretory)	[15]
GM-CSF	T/tobacco cells suspension	35S promoter with the double enhancer, *nos* terminator	180–780 μg/L	[16]
GM-CSF	M/rice cells suspension	rice amylase promoter and signal peptide	129 mg/L (25% secreted proteins)	[17]
GM-CSF	M/rice cells suspension rice	amylase promoter and signal peptide, RNAi-mediated silencing of α-amylase gene to 8.2%	280 mg/L	[18]
GM-CSF	M/rice cells suspension	rice amylase promoter and signal peptide, RNAi-mediated silencing of cysteine proteinase	290 mg/L	[19]
GM-CSF	M/rice cells suspension	rice amylase promoter and signal peptide; co-expression of gene encoding synthetic protease inhibitor (SPI-II)	250 mg/L	[20]
GM-CSF	M/sugarcane leaves	*MUbi-1* promoter from maize or *SCubi-9* from sugarcane	0.02% TSP	[21]
GM-CSF	T/tobacco seeds	*Gt1*, *Gt3* (glutelin) promoters and signal peptide, *nos* terminator	0.005–0.03% TSP	[22]
GM-CSF	T/tobacco seeds	*Gt1* (glutelin) promoter and signal peptide, *nos* terminator	1.3% TSP	[23]
GM-CSF	T/rice seeds	*Gt13a* (glutelin) promoter (specific for seed endosperm) and glutelin signal peptide, *nos* terminator, codon optimalization	0.5–14 μg/seed	[24]
Murine GM-CSF	T/tobacco leaves	*RbcS1* Promoter; signal peptide, KDEL	19 μg/g fresh leaves; 0.22%	[25]
GM-CSF	V/*N. benthamiana* leaves	PVX-derived vector: 35S promoter, His tag	0.2–2% TSP	[26]
IL-2	T/tobacco cells suspension	35S promoter, T7 terminator	0.09 mg/L	[27]
IL-2	T/potato tubers	patatin promoter; *nos* terminator	115 U/mg TSP	[28]
Murine IL-2	T/Arabidopsis seeds T/tobacco seeds	novel binary Gateway vector (pPphasGW) containing β-phaseolin promoter from common bean and the signal peptide of the Arabidopsis 2S2 seed storage protein gene; KDEL	Much higher yield in Arabidopsis than in tobacco: 0.3 mg/g of seeds (0.7% TSP); biologically active *in vitro*	[29]
IL-4	T/tobacco leaves	35S promoter with double enhancer, t-CUP-translation enhancer, ELP, KDEL, *nos* terminator	0.086% TSP	[30]
IL-4	T/tobacco leaves, T/potato tubers	35S promoter, KDEL sequence	0.1% TSP in tobacco; 0.08% TSP in potato	[31]
IL-4	T/tobacco cells suspension	35S CaMV promoter, T7 terminator	0.45 mg/L	[27]
IL-10	T/tobacco leaves	35S promoter with double enhancer, t-CUP-translation enhancer, ELP, KDEL, *nos* terminator	0.27% TSP	[30]
IL-10	T/tobacco leaves	promoter 35S CaMV, with or without His tag, chloroplast leader peptide promotor 35S CaMV, His tag; itochondria leader peptide	7 ng/mg TSP (without His); 43 ng/mg TSP (with His) no accumulation	[32]
IL-10	T/rice seeds	GluB-1 promoter and signal peptide, His tag, KDEL, codon optimization	2 mg of pure IL-10 per 40 g of rice powder	[33]
IL-10	T/tobacco leaves	35S promoter with double enhancer; three constructs for each viral IL-10 or murine IL-10, ER-targeted, plasma membrane (IL-10 facing the apoplast), ER-membrane (IL-10 facing the cytosol) assayed in transient expression system, cassettes for ER-targeted cytokines were used for the stable expression	Viral: 10.8 μg/g fresh leaves Murine: 37.0 μg/g fresh leaves	[34]
IL-12	T/tobacco leaves	35S promoter and terminator	40 ng/g	[35]
IL-12	T/tomato leaves and fruits	35S promoter with double enhancer; 35S terminator	7.3 μg/g leaves 4.3 μg/g fruits	[36,37]
IL-12	T/tobacco cells suspension	35S promoter with double enhancer; Ω-translation enhancer	175 μg/L	[38]
IL-13	T/tobacco leaves	double 35S promoter; translation enhancer from AMV; KDEL; *nos* terminator	0.15% TSP	[39]
IL-18	T/tobacco leaves	35S promoter with double enhancer, Ω-translation enhancer, *nos* terminator	0.004–0.051% TSP; 351 ng/g	[40]
cardiotrophin-1	M/tobacco leaves, chloroplasts transformation	promotor *rrn* (promoter 16S RNA), translation enhancer (5′UTR/leader sequence of the phage T7 gene 10) *psbA* promoter and translation enhancer (5′UTR *psbA*)	0.14 mg/g leaves up to 1.14 mg/g leaves	[41]
IFN-α2b IFN-α8	T/potato	-	560 IU/g of tissue	[42]
IFNα	L/tomato (leaf tissue and cells suspension)	P1 portion of the dual “bi-directional” promoter from *A. tumefaciens* cDNA, polyadenylation signal from *A. tumefaciens* gene-7	923–3029 U/g FW tissue	[43]
IFN-α2	V/squash (*Cucurbita pepo*) and cucumber (*Cucumis sativus*)	Viral vector dirived from attenuated zucchini yellow mosaic potyvirus (AG)	max. 430,000 IU/FW of leaves	[44]
IFN-α2b	M/tobacco leaves, chloroplasts transformation	Cassette: 5′UTR/HIS/THR/IFNα2b cloned into the chloroplast vector pLD-CtV	3 mg/g, 20% TSP	[45]
IFN-α2b	T/carrot leaves	35S promoter, *nos* terminator, calreticulin apoplast targeting signal taproot-specific Mll promoter, *nos* terminator, calreticulin apoplast targeting signal	Biological activity on average: 26.8 × 10^3^ U/g FW of young leaves 8.56 × 10^3^ U/g FW of roots	[46]
IFNβ	transient, agroinfiltration of the leaves of lettuce	35S promoter, *nos* terminator	3.1 × 10^4^ IU/mL	[47]
IFNγ	T/rice cells suspension	constitutive maize ubiquitin promoter, with or without αAmy3 leader peptide; His tag; sucrose-starvation inducible promoter (rice αAmy3 promoter); with or without αAmy3 leader peptide; His tag	11.1 ng/mL (secretory) and 699.79 ng/g cell (intracellular) 17.4 ng/mL media (secretory) and 131.6 ng/g cell (intracellular)	[48]
Chicken IFN-α	Transient expression, agroinfiltration of the leaves of lettuce	35S promoter, *nos* terminator	0.393 μg/kg tissue, 0.0004% TSP	[49]
Fish IFN-α1	T/rice T/potato	35S promoter with double enhancer, *nos* terminator	Biological activity: in rice-up to 0.82 U/mg leaves; in potato-up to 5.4 U/mg leaves	[50]
TNF-α	T/potato	35S promoter, Ω-translation enhancer, SEKDEL sequence	15 μg/g tissue	[51]
Fibroblast growth factor 8 isoform b (FGF8b)	T/tobacco leaves	35S promoter with double enhancer; 35S terminator, c-myc, His, KDEL	4.1% TSP	[52]
Insulin like growth factor 1 (IGF-1)	M/tobacco, transplastomic	psbA promoter, translantion enhancer (5′UTR psbA) and ZZ-tag from *S. aureus*, codon optimization	up to 32% TSP	[53]
Insulin like growth factor 1 (IGF-1)	M/rice seeds	Glutelin (*Gt13a*) promoter, the *Gt13a* signal peptide in frame with the fusion protein containing IGF-1 attached to the *C*-terminus of ER luminal binding protein (BipC), *nos* terminator	up to 6.8% of total seed protein; biologically active *in vivo* (effectively reduced blood glucose in diabetic mice)	[54]

Different expression systems are marked differently (no background-suspension cultures; light grey-transgenic or transplastomic plants; dark grey-plant virus-based production); *nos*-nopaline transcriptional terminator, MUbi-1 i Scubi-9-polyubiquitin promoters;

* Method of transformation: T-Agrobacterium-mediated; M-microbombardment, V-viral vector, l-lipofectin-mediated transformation of protoplasts.

The examples concern human cytokines unless indicated otherwise.
